# Impact of the flavonoid-induced intestinal microbiota modulation on global energy metabolism: implication for obesity

**DOI:** 10.3389/fnut.2025.1696152

**Published:** 2026-01-02

**Authors:** Omar Guzmán-Quevedo, Alana Natalícia Vasconcelos de Araújo, Pedro A. Romero-Juárez, Jordania Candice Costa Silva, Gonzalo Soria-Melgarejo, Luz Torner, Jailane de Souza Aquino

**Affiliations:** 1 Laboratory of Experimental Neuronutrition and Food Engineering, Tecnológico Nacional de México (TECNM)/Instituto Tecnológico Superior de Tacámbaro, Tacámbaro, Michoacán, Mexico; 2 Centro de Investigación Biomédica de Michoacán, Instituto Mexicano del Seguro Social, Morelia, Michoacán, Mexico; 3 Postgraduate Program in Neuropsychiatry and Behavioral Sciences, Federal University of Pernambuco, Recife, PE, Brazil; 4 Laboratory of Experimental Nutrition, Department of Nutrition, Federal University of Paraíba (UFPB), João Pessoa, PB, Brazil; 5 Post Gradutate Program of Nutrition Sciences, Federal University of Paraíba (UFPB), João Pessoa, PB, Brazil; 6 Tecnológico Nacional de México (TECNM)/Instituto Tecnológico Superior Purépecha, Cherán, Michoacán, Mexico; 7 Post Graduate Program of Food Science and Technology, Federal University of Paraíba (UFPB), João Pessoa, PB, Brazil; 8 Tecnológico Nacional de México (TECNM)/Instituto Tecnológico Superior de Puruándiro, Puruándiro, Michoacán, Mexico

**Keywords:** energy metabolism, flavonoids, inflammation, intestinal microbiota, obesity

## Abstract

Evidence from the literature clearly demonstrates the beneficial effects of flavonoids on energy metabolism. Due to this, they have become important candidates for combating metabolic disorders like obesity and diabetes. Intestinal microbiota (IM) has shown similar effects on metabolic regulation, contributing to host health. Several studies have shown concomitant effects on metabolism and intestinal microbial profile in response to flavonoid-based treatments. However, the role of the bidirectional interaction between IM and flavonoids in the metabolic effects of these organic compounds is less well-established. This review discusses the effects of flavonoids on the IM in regulating energy homeostasis in metabolically relevant tissues, including skeletal muscle, liver, adipose tissue, and hypothalamus. The modulation of the gut microbiota by dietary flavonoids and *vice versa* is also discussed. Understanding the contribution of each actor in this interaction, as well as their mechanisms of action, can help design dietary and nutritional strategies to combat metabolic disorders, including obesity, type 2 diabetes, and dyslipidemia.

## 1 Introduction

**Figure 1:**
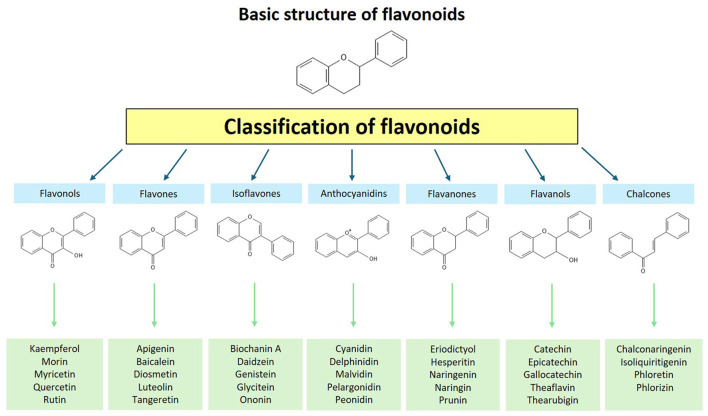
Classification of flavonoids.

Flavonoids, belonging to the group of polyphenolic compounds, are secondary metabolites produced by plants. Based on the degree of unsaturation and oxidation of their carbon rings, flavonoids are generally divided into seven classes: flavonols, flavones, isoflavones, anthocyanidins, flavanones, flavanols, and chalcones. In turn, each class is composed of a significant number of compounds (Figure 1) (1). With a high presence in a wide range of fruits and vegetables, flavonoids exhibit various beneficial biochemical effects such as anti-inflammatory, anti-aging, and hypoglycemic actions (2, 3). The beneficial effects of flavonoid consumption on different components of metabolic syndrome have been demonstrated in clinical studies, demonstrating suitability to address metabolic disorders (4). The term “phytonutrient” has been assigned to them due to the essential role of flavonoids in metabolic health.

Intestinal microbiota (IM) refers to the set of microorganisms that colonize the gastrointestinal tract, particularly the lumen of the intestinal tract (5). This exhibits extreme diversity and variability among individuals, consisting mainly of four primary phyla: *Bacteroidetes, Firmicutes, Actinobacteria*, and *Proteobacteria* phyla. The most common phyla are *Bacteroidetes* and *Firmicutes*, accounting for ~90% of the species (6). These microorganisms offer various health benefits related to physiological functions, such as the immune system and metabolic regulation, because of evolution and long-term interaction with the host (7). IM is composed of an intricate diversity of microorganisms, including bacteria, fungi, archaea, and microeukaryotes. Thus, its composition can be easily altered by several factors, such as unhealthy diets, genetics, the use of medication and antibiotics, the presence of pathogens, and physical exercise (8, 9). This imbalance is known as dysbiosis, which refers to an irregular degree of IM variability. Prolonged dysbiosis can cause interferences with homeostasis, leading to local and systemic inflammatory events and altered responses, which can result in metabolic disorders (10). Regarding energy homeostasis, IM plays an important role through the secretion of several messengers that impact key processes in the regulation of energy metabolism (11).

Although the effects of both flavonoids and IM on energy metabolism are well-established, the impact of the interaction between these two metabolic actors on the organism's energy status is less clear. In this context, this review discusses experimental evidence showing the mechanisms involved in the IM regulation by flavonoids and its impact on energy homeostasis. The review focuses on metabolically active tissues, including skeletal muscle, liver, adipose tissue, and hypothalamus, a key player in energy balance (EB) regulation. We also discuss the mutual regulation between flavonoids and IM.

## 2 Metabolic actions induced by flavonoids

Due to the overwhelming prevalence of metabolic diseases, flavonoids have attracted great attention for their ability to influence energy metabolism. Their wide range of metabolic activities include the regulation of insulin secretion, glucose and lipid metabolism, and inflammation, a physiological state closely linked to metabolic diseases (4, 12). Preclinical studies recently conducted, demonstrating the antiglycemic and antidyslipidemic effects of several flavonoids, are presented below.

Hypoglycemic properties have been documented with the use of several flavonoids including, kaempferol, myricetin, naringin, catechins, and sudachitin. Treatment with kaempferol (50 mg/kg/day) for 6 weeks increased glucose tolerance and insulin sensitivity, along with body weight (BW) loss in obese (*db*/*db*) mice with insulin resistance (13). The study showed that these effects are due to a decrease in the adipose tissue inflammation involving the STING/NLRP3 signaling pathway (13). In another study, streptozotocin-induced diabetic rats exposed to a high-fat diet (HFD) and treated with myricetin showed reduced plasma glucose and insulin levels. These effects were likely due to increased expression of the insulin receptor (IR) and the glucose transporter 4 (GLUT4) (14). The effectiveness of this flavonoid can be seen even at low doses, as the effects were observed with doses of 50 and 200 mg/kg. Consumption of catechins from green tea has been associated with the regulation of intestinal immunometabolic homeostasis. The treatment with these flavonoids decreased mucosal inflammation, improved intestinal barrier function, and mitigated gut dysbiosis, favoring improved glucose metabolism in extraintestinal tissues (15). Similarly, naringin and naringenin improved insulin sensitivity by activating GLUT4 and peroxisome proliferator-activated receptor-γ (PPAR-γ). They also showed antioxidant and anti-inflammatory effects through the modulation of the mitogen-activated protein kinase (MAPK), nuclear factor kappa-light-chain-enhancer of activated B cells (NF-κB), and nuclear factor erythroid 2-related factor 2 (Nrf2) pathways. It makes them potential therapeutic compounds for diabetes *mellitus* and its complications (16). Finally, sudachitin has been shown to double insulin secretion in high glucose concentration conditions in mouse pancreatic islets and mouse insulinoma cell line (MIN6) (17). Oral administration enhances early insulin secretion and reduces blood glucose by 30% after a glucose load. This effect is due to the potent inhibitory effect (half maximal inhibitory concentration inhibition -IC_50_- around 10 μM) of sudachitin on phosphodiesterase (PDE) (17).

Regarding lipid metabolism, several flavonoids have shown significant benefits. In experimental models, quercetin increased the expression of the adiponectin receptor (AdipoR2) in the liver and heart, promoting fatty acid oxidation through peroxisome proliferator-activated receptor-α (PPAR-α) (18). Conversely, reduced hepatic lipid synthesis was observed by decreasing sterol regulatory element-binding protein 1c (SREBP-1c) and fatty acid synthase (FASN), suggesting an anti-obesity and cardioprotective effect (18). In the same line, kaempferol promoted the formation of beige adipocytes through the activation of AMP-activated protein kinase (AMPK) (19). This effect involves the expression of uncoupling protein 1 (UCP1), sirtuin 1 (SIRT1), and peroxisome proliferator-activated receptor gamma coactivator 1 alpha (PGC-1α), promoting energy metabolism and thermogenesis (19). Consistent with the previous results, myricetin, a flavonol like quercetin and kaempferol, and its glucoside myricitrin showed anti-inflammatory effects. Treatment with these compounds reduced the production of tumor necrosis factor α (TNF-α) and interleukin-1β (IL-1β), restoring inflammation-induced inhibition of UCP1 expression in beige adipocytes (20). It suggests their usefulness in promoting thermogenesis by adipocyte browning. The effect of flavonoids on lipid metabolism is not restricted to flavonols. Treatment with catechins, members of the flavanol group, resulted in a decrease in GLUT4 expression and an increase in UCP1 expression in differentiated 3T3-L1 adipocytes, which indicates a browning process (21). Finally, the treatment with luteolin, a flavone, inhibited adipogenic differentiation by reducing reactive oxygen species and inhibiting adipogenic transcription factors, improving cellular redox status (22). This study found that quercetin, myricetin, apigenin, kaempferol, and chrysin reproduce the antiadipogenic effects of luteolin, suggesting that the anti-obesity effects are a feature of flavonoids (22).

Overall, the data presented in this section show high reproducibility regarding the antihyperglycemic and antidyslipidemic effects of flavonoids. The hypoglycemic effects induced by flavonoids generally involve an increase in insulin sensitive (increased IR expression), GLUT4, and PPAR-γ. A reduction in inflammation in adipose tissue also restored insulin sensitivity. Regarding the regulation of lipid metabolism, flavonoids activate adiponectin- and PPAR-α-dependent signaling pathways, promoting fatty acid oxidation. In adipocytes, they promote thermogenesis and browning processes through the induction of UCP1 activity and the activation of AMPK-, SIRT1-, and PGC-1α-dependent signaling pathways. Anti-inflammatory effects were also implicated. A point to highlight is the fact that the metabolic benefits are independent of the classes of flavonoids, indicating that the basic chemical structure of these compounds underlies their effects. This may explain why simple (myricetin) or glycosylated (myricitrin) forms exhibit beneficial effects (20). This is of utmost relevance considering the biotransformation processes that flavonoids undergo when ingested.

## 3 Metabolic actions induced by intestinal microbiota

**Figure 2:**
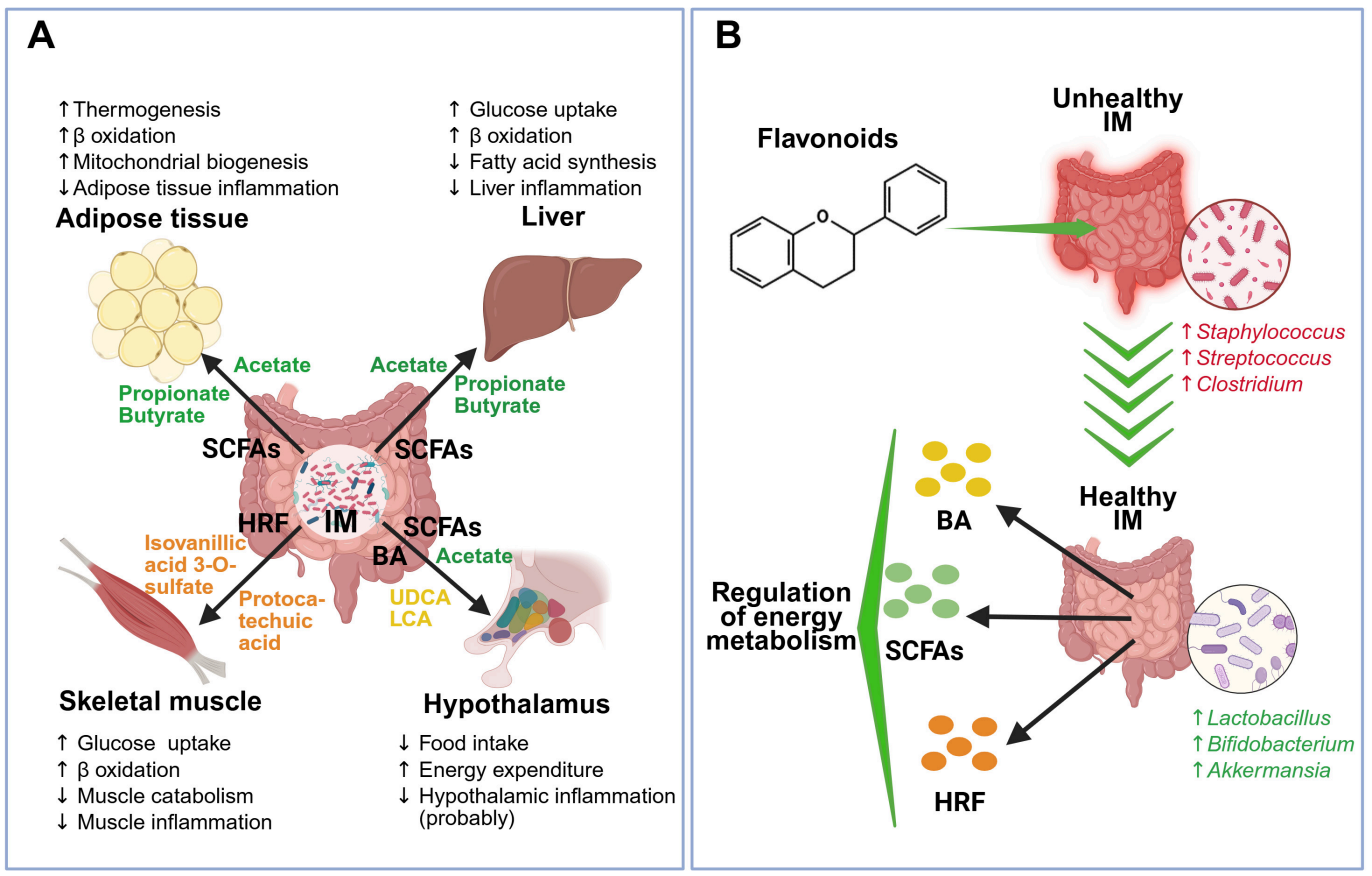
Effects of intestinal microbiota and flavonoids on energy metabolism. **(A)** Effects of intestinal microbiota (IM) on energy metabolism in adipose tissue, liver, skeletal muscle, and hypothalamus. IM produces different messengers, including short-chain fatty acids (SCFAs), bile acids (BA), and hydrolyzed and reduced flavonoids (HRFs). SCFAs (acetate, propionate, and butyrate) induce an increase in thermogenesis, β oxidation, and mitochondrial biogenesis, as well as a decrease in inflammation in adipose tissue. In liver, SCFAs increase glucose uptake and β oxidation and decrease fatty acid synthesis and liver inflammation. In hypothalamus, the SCFA acetate decreases food intake. BAs, ursodeoxycholic acid (UDCA) and lithocholic acid (LCA), promote hypothalamic energy expenditure and decrease inflammation in this tissue. In skeletal muscle, HRFs (isovanillic acid 3-O-sulfate and protocatechuic acid) increase glucose uptake, β oxidation, and decrease muscle catabolism and muscle inflammation. **(B)** Flavonoids switch unhealthy IM (characterized by excessive abundance of *Staphylococcus, Streptococcus*, and *Clostridium* bacteria) to healthy IM (characterized by abundant *Lactobacillus, Bifidobacterium*, and *Akkermansia* bacteria), which produces compounds such as BA, SCFAs, and HRF, restoring the regulation of energy metabolism. Created with BioRender.com.

IM plays a key role in metabolic regulation through its effect on different organs (Figure 2A). These intestinal microorganisms collaborate at different levels by supplying nutrients, metabolites, and usable energy, targeting distinct sites of action and functions. Such actions are carried out by breaking down dietary components to produce metabolites, modifying host-derived metabolites, and even synthesizing new metabolites (23). Regarding the breakdown of compounds by IM and their conversion into beneficial or harmful metabolites, the activation of different metabolic pathways is required to break down indigestible dietary fibers into beneficial short-chain fatty acids (SCFAs) (24). A specialized network of primary and secondary degraders is activated, functioning in a cycle where the waste of one group of microorganisms becomes a substrate for others. Fibers such as cellulose and pectin can undergo bacterial fermentation, generating final metabolic products such as acetate, propionate, and butyrate, which are recognized energy generators and beneficial modulators of the immune system (25).

Another highly relevant action of IM is its association with the consumption and production of micronutrients. In this dual effect, on one hand, the microorganisms present in the host's microbiota consume vitamins and minerals from the diet for their survival and functionality. This allows, for example, the association of certain vitamin supplements (vitamins A, C, D) with microbiota composition. On the other hand, IM can participate in the production of a wide range of vitamins, particularly B-complex vitamins and vitamin K, as well as facilitate the absorption of minerals like iron and calcium (26).

IM also participates in the degradation of diet-derived proteins, particularly rich in tryptophan, an essential amino acid that can be metabolized and converted into various metabolites related to indole signaling. These metabolites have an impact on intestinal homeostasis, immunity, and the development of conditions such as obesity, inflammatory bowel diseases, metabolic syndrome, tumors, cardiovascular diseases, nervous system disorders, and liver fibrosis (27). In the synthesis of endogenous substances, IM is involved in the metabolism and production of bile acids. Secondary bile acids are produced by IM from primary bile acids synthesized in the liver and released into the intestine through the enterohepatic cycle. Microorganisms of the *Lactobacillus* genus promote the production of secondary bile acids, which influence the regulation of energy metabolism (28, 29).

From what we know so far, it can be said that the gut microbiota, through the production of metabolites such as bile acids, SCFAs, neurotransmitters, etc., plays a crucial role in energy homeostasis (Figure 2A). These metabolites can impact different organs such as the hypothalamus, liver, adipose tissue, and skeletal muscle, regulating different energy metabolism pathways (11). The details of these mechanisms will be discussed in the next sections.

## 4 Mutual regulation between flavonoids and intestinal microbiota

In addition to the mechanisms mentioned above, there are beneficial host-diet interactions in which IM, involving flavonoids, plays a central role (Figure 2B). Flavonoids can exert prebiotic effects on IM, enabling control of pathogenic populations and promoting symbiosis. In the opposite direction, IM exerts chemical transformation on flavonoids, facilitating their absorption, increasing bioavailability and functionality. This interaction is beneficial in different metabolic disorders, including obesity, cardiovascular diseases, and diabetes (30).

Flavonoids are present in various food matrices. When ingested but not absorbed, or when their metabolites reach the colon, they interact with the microbiota, modulating its population and generating multiple effects through various mechanisms of action (1, 30, 31). Flavonoids are selectively absorbed in the gastrointestinal tract following oral administration. While some hydrophobic compounds, such as aglycones, can be absorbed in the stomach, others, such as flavonoid glycosides, are not. Some flavonoids are absorbed in the small intestine, whereas others are subjected to enzymatic hydrolysis or reduction by IM enzymes, converting them into chain catabolites or monomers with increased absorption and bioactivity (32). These are then transferred through specific channels, exiting the intestine and reaching various organs. This complex cycle of microbial transformations generates an intricate metabolic network, culminating in changes in both microbiota and host metabolic health (32, 33).

Synthetic and food-derived flavonoids are being evaluated for their antimicrobial properties, given the current demand for functional foods and new treatments for infectious diseases, as human pathogens are becoming increasingly resistant to existing antibiotics. A study examined the antimicrobial activity of 12 chemically synthesized flavonoid molecules (four chalcones, four flavones, and four flavanones) against nine Gram-negative and Gram-positive bacterial strains and three fungal strains (34). The study demonstrated moderate to high antibacterial activity against Gram-negative (*Escherichia coli*-*E. coli*-, *Salmonella spp*., *Pseudomonas aeruginosa*) and Gram-positive (*Enterococcus faecium, Bacillus cereus*) strains, with particularly strong effects against *Staphylococcus aureus* (*S. aureus*) (34). Regarding the antifungal activity, chalcones were the most potent compounds, followed by flavanones and flavones, against *Aspergillus niger, Aspergillus flavus*, and *Penicillium expansum* (34). Additionally, the antibacterial properties of three flavonoid extracts from medicinal plants (*Achillea millefolium, Bergenia ciliata*, and *Aloe vera*) were evaluated. All the extracts studied had significant antibacterial effects against *S. aureus* and *E. coli* (35). These findings highlight their potential for pharmaceutical applications to modulate IM in the context of metabolic syndrome, as flavonoids preferentially eliminate pathogenic microorganisms.

Active microbiota modulation and bidirectional modification can also protect the intestinal barrier. This barrier serves as a defense mechanism, separating the luminal environment and preventing pathogenic bacteria, harmful substances, and toxins from reaching host cells. It also restricts the movement of endotoxins and bacteria inherent to this environment, thereby preserving intestinal health. The protective effect of flavonoids on the microbiota and intestinal barrier can be attributed to various mechanisms. These include permeability regulation, inhibition of inflammatory signaling pathways, immune modulation, reduction of oxidative stress in the intestinal lumen, promotion of structural integrity, and improved barrier function through upregulation of glucagon-like peptide-2 (GLP-2) (32).

Preclinical and clinical studies using flavonoid sources have reported modulation of IM along with effects reflecting metabolic benefits. Chen et al. (36) examined the impact of green tea polyphenols on the intestinal microbiota and diabetes development in *db*/*db* mice. The study revealed significant changes in bacterial communities, including a reduction in *Firmicutes* and *Bacteroidetes*, associated with antidiabetic effects. Additionally, Lima et al. (37) carried out a clinical study in women and found that polyphenols from orange juice consumption increased the populations of *Bifidobacterium* and *Lactobacillus* while suppressing metabolic disorders. In line with this, a study evaluated the influence of seabuckthorn berry juice in an *in vitro* intestinal model, observing a stimulating effect on beneficial microbial populations such as *Lactobacillus, Bacteroides*/*Prevotella*, and *Bifidobacteria* (38). The authors also reported a high release of polyphenols and an increase in antioxidant activity (38). A relevant fact is that although the studies were carried out in mice, humans, and an artificial intestine, similar effects in response to treatment with polyphenols were observed; that is, the promotion of a healthy microbiota.

The evidence presented in this section clearly shows a bidirectional regulation between flavonoids and IM with a significant effect on metabolic status. On the one hand, flavonoids regulate intestinal bacterial populations, promoting a favorable microbial profile. On the other hand, resident intestinal bacteria biotransform flavonoids, also inducing metabolic effects. Considering this, it is important to design preclinical and clinical studies focused on determining the most appropriate flavonoid intervention based on the individual's microbiota composition. Given the high diversity of microorganisms and flavonoids, the challenge is to generate evidence allowing us to move toward personalized flavonoid-based treatments.

## 5 Metabolic effects on skeletal muscle induced by the interaction between flavonoids and intestinal microbiota

Skeletal muscle plays a crucial role in glucose uptake and the regulation of energy metabolism (39, 40). The interaction between flavonoids and IM has been recognized as a key factor in modulating muscle metabolism and functionality (Table 1) (41, 42). The biotransformation of flavonoids by the gut microbiota generates bioactive metabolites that can modulate the insulin signaling pathway, stimulate mitochondrial biogenesis, reduce inflammation, and regulate the production of SCFAs in muscle tissue. Studies on the role of phenolic compounds in the gut-muscle axis have primarily focused on their effects under catabolic conditions, such as sarcopenia and muscle damage, or under anabolic conditions, particularly in response to exercise (42, 43).

**Table 1:** Skeletal muscle responses to flavonoid-mediated microbiota modulation.

**Study type**	**Biological model**	**Compounds**	**Method/dose**	**Effects**	**References**
*In vitro*	Differentiated human skeletal muscle myoblasts (LHCN-M2 cell line)	Isovanillic acid 3-O-sulfate	Tested at concentrations ranging from 0.1 to 10 μM	Stimulated a dose-dependent increase in glucose transport through GLUT4 and PI3K-dependent mechanisms; ↑ GLUT1, GLUT4, and PI3K protein expression; ↑ phosphorylation of AKT.	(46)
Animal model	Female C57BL/6 mice (old: 22–26 months old)	Hesperidin	Oral administration, 5 or 10 mg/kg/day for 8 weeks	↑ Muscle mass and strength; enlarged muscle fiber size; modulated AKT/mTOR/FoxO3a signaling pathway.	(48)
Clinical	Amateur competitive cyclists	2S-Hesperidin	Double-blind, parallel, randomized controlled trial 500 mg/day, oral supplementation for 8 weeks	↓ Fat mass; ↑ muscle mass percentage (1%); ↑ total muscle mass (1.7%).	(49)
Animal model	C57BL/six mice with high-fat diet-induced obesity	Luteolin	Oral supplementation, 50 mg/kg/day for 12 weeks	Suppressed lipid infiltration in the gastrocnemius muscle; ↓ inflammatory markers (TNF-α, TLR2, TLR4, MCP1, MMP2); attenuated muscle protein degradation.	(54)
Animal model	Ovariectomized female C57BL/6J mice	Isoflavone	0.1% isoflavone-supplemented drinking water for 6 weeks	↑ Grip strength; decreased expression of muscle atrophy-related genes; altered gut microbiota composition (with an increase in *Bacteroides* and CAG5); ↑ serum concentrations of equol and daidzein.	(59)
*In vitro*	C2C12 myotubes	Daidzein	Tested at concentrations ranging from 0.1 to 10 μM	↓ Lipid accumulation in muscle cells; ↑ oxygen consumption; ↑ genes related to oxidative phosphorylation; fatty acid oxidation via the ERRα pathway.	(60)
Animal model	Male mice	Protocatechuic acid	Dietary supplementation with 50 or 100 mg/kg for 28 days	↑ Expression of slow MyHC; ↓ expression of fast MyHC; ↑ antioxidant capacity; promoted mitochondrial biogenesis; activated AMPK signaling pathway.	(64)

↑, increase; ↓, decrease; AKT, protein kinase B; AMPK, AMP-activated protein kinase; ERRα, estrogen-related receptor alpha; FoxO3a, Forkhead box O3a; CAG5, co-abundant gene group; GLUT, glucose transporter; MCP1, monocyte chemoattractant protein-1; MMP2, matrix metalloproteinase 2; mTOR, mammalian target of rapamycin; MyHC, myosin heavy chain; PI3K, phosphoinositide 3-kinase; TLR, toll-like receptor; TNF-α, tumor necrosis factor α.

Flavonoids, through their interaction with IM, can enhance insulin-stimulated glucose uptake in skeletal muscle, thereby promoting muscle performance (44). This modulation occurs via the microbial conversion of these compounds into bioactive metabolites, which influence metabolic pathways associated with glycemic homeostasis (45). Among these metabolites, the flavonoid isovanillic acid 3-O-sulfate found in berries, derived from cyanidin-3-O-glucoside, has been shown to induce a dose-dependent increase in glucose uptake in differentiated human skeletal muscle myoblasts (LHCN-M2 cell line) (46). This effect was mediated by mechanisms dependent on GLUT4 and the phosphoinositide 3-kinase (PI3K) signaling pathway, reinforcing its role in the regulation of insulin sensitivity and the metabolic function of skeletal muscle (46). The use of cell lines is very useful for elucidating the molecular mechanisms involved in the metabolic effects of flavonoid-derived metabolites. However, *in vivo* assessments under physiological conditions are necessary to determine the gut microbiota profile required to obtain the metabolite being tested, as well as the reproducibility of the actions of isovanillic acid 3-O-sulfate in a complex system.

Flavanones are widely distributed in citrus fruits and are recognized for their antioxidant and metabolism-modulating properties (47). Among these compounds, hesperidin is one of the most extensively studied. In aged female mice, hesperidin supplementation for 8 weeks significantly increased muscle mass, strength, and muscle fiber size (48). Furthermore, hesperidin was found to attenuate sarcopenia by regulating the AKT (protein kinase B)/mTOR (mammalian target of rapamycin)/FoxO3a (forkhead box O3a) signaling pathway, leading to an increased expression of insulin-like growth factor (IGF-1), which is essential for muscle growth and maintenance (48). In a double-blind randomized clinical trial, involving 40 amateur cyclists, hesperidin supplementation for 8 weeks resulted in a notable increase in muscle mass (49). It is known that the bioavailability of hesperidin in the intestinal tract is limited, which may compromise its systemic efficacy. For absorption by the intestinal mucosa, hesperidin must be converted into its active form, hesperetin. This biotransformation is mediated by the intestinal microbiota, particularly by bacteria of the genus *Bifidobacterium*, with *Bifidobacterium pseudocatenulatum* playing a crucial role in this process (50, 51). The studies above did not determine the composition of the microbiota, so it remains to be proven whether hesperidin exerted its effects directly or involved bacterial metabolism to produce hesperetin.

The interaction between flavonoids and the IM can reduce the activation of the inflammatory system, creating a metabolic environment more favorable to muscle function (52). Flavonoid-derived metabolites exert anti-inflammatory effects by modulating inflammatory cytokines and reducing oxidative stress in skeletal muscle, thereby contributing to the maintenance of muscle mass and function (52, 53). Supplementation with luteolin, a flavonoid from the flavone class, has been shown to suppress lipid infiltration in the gastrocnemius muscle (54). Such an effect was associated with reduced inflammatory markers, including TNF-α, toll-like receptor 2 (TLR2), TLR4, monocyte chemoattractant protein 1 (MCP1), and matrix metalloproteinase (MMP2), in mice with sarcopenic obesity (54). These effects resulted in reduced protein degradation and improved muscle function (54). The metabolic pathways of flavones remain less understood compared to other subclasses of flavonoid compounds. The bioavailability of these compounds is largely dependent upon the microbial hydrolysis process, which is carried out by only a select few microorganisms such as *Enterococcus avium, Parabacteroides distasonis, Eubacterium ramulus* (*E. ramulus*), and *Flavonifractor plautii* (42, 55, 56). This process leads to the formation of a variety of absorbable bioactive compounds, which may positively influence muscle homeostasis and inflammatory modulation. Although luteolin metabolites produced by IM may be involved, it cannot be ruled out that luteolin exerts its effects directly on muscle. It has been shown to be rapidly absorbed, reaching high plasma concentrations 1 h after administration in rats (57).

Isoflavones are flavonoid compounds with a molecular structure like that of human steroidal estrogens. When consumed, they can exert either estrogenic or anti-estrogenic effects (58). The administration of isoflavones to ovariectomized mice led to an increase in the abundance of beneficial bacteria, particularly from the genera *Bacteroides* and CAG5 (co-abundant gene group 5) (59). The modulation of the IM was accompanied by an increase in serum concentrations of equol and daidzein, key metabolites derived from isoflavone metabolism (59). In addition to changes in the microbiome, suppression of gene expression related to muscle atrophy was observed, suggesting a protective effect against protein degradation in skeletal muscle (59). Also, the activation of the TNF-α/NF-κB inflammatory pathway, which is linked to muscle catabolism, decreased, thus creating a more favorable metabolic environment for maintaining muscle mass (59). In another study, daidzein was found to promote oxidative phosphorylation and fatty acid oxidation in murine muscle cells via an estrogen-related receptor alpha (ERRα)-associated pathway. This resulted in a reduction in lipid deposition in muscle tissue (60).

IM's biotransformation of flavonoids results in metabolites capable of activating PGC-1α and AMPK pathways, which are associated with enhanced mitochondrial biogenesis and improved muscle function (61, 62). For quercetin to have biological activity, it must undergo metabolic conversion. Its biotransformation in the intestine by bacterial strains such as *Bacteroides fragilis, E. ramulus*, and *Clostridium perfringens* produces bioactive metabolites, including protocatechuic acid (63). Yang et al. (64) demonstrated that the administration of protocatechuic acid to mice promoted mitochondrial biogenesis in the gastrocnemius muscle and converted type II (fast) to type I (slow) skeletal muscle fibers. This effect was mediated by the activation of the AMPK signaling pathway, promoting a more oxidative, fatigue-resistant, and energy-efficient muscle profile (64).

Another mechanism by which flavonoids and their metabolites may influence skeletal muscle is through the regulation of SCFA production by the gut microbiota (32). Among the most abundant SCFAs are acetate, propionate, and butyrate, which play essential roles in metabolic homeostasis and muscle function (65). It has been proven by studies that consumption of phenolic compounds, such as anthocyanins, quercetin, and catechins, stimulates the growth of bacteria producing SCFAs, including *Bifidobacterium* spp., *Faecalibacterium prausnitzii*, and *Lactobacillus* spp. (66–69). The production of these microbial metabolites can directly influence muscle metabolism through the activation of G protein-coupled receptors (GPCRs), such as free fatty acid receptor 3 (FFAR3) and free fatty acid receptor 2 (FFAR2), expressed in muscle tissue (70, 71).

The evidence presented above demonstrates the bidirectional relationship between flavonoids and skeletal muscle metabolism. The clearest mechanisms in these effects primarily involve bacterial production of flavonoid metabolites, the production of SCFAs, and the generation of an anti-inflammatory environment. Several studies investigated the effects of flavonoids or their metabolites on skeletal muscle metabolism. However, they did not measure the gut microbiota profile to establish its causal role or at least a correlation with the observed effects. Future research is necessary to fix these gaps. Since the metabolism of this tissue has a significant impact on EB and BW, therapies targeting the activation of the flavonoid-IM interaction represent an opportunity to counteract metabolic diseases.

## 6 Impact of the interaction between flavonoids and intestinal microbiota on hepatic energy metabolism

The metabolic interaction between the liver and intestine is mediated by a complex flux of bidirectional communication known as the gut-liver axis. This relationship plays a fundamental role in maintaining systemic energy homeostasis and is strongly influenced by the composition and activity of IM (72). Intestinal microorganisms convert flavonoids into metabolites that have greater bioavailability and enhanced biological activity, which have beneficial effects on liver function (Table 2) (73). These metabolites regulate key hepatic metabolic pathways, influencing processes such as gluconeogenesis, fatty acid β-oxidation, and glycogen synthesis (74–76). Thus, the interplay between flavonoids, the intestinal microbiota, and hepatic metabolism emerges as a crucial mechanism in the modulation of energy metabolism and the prevention of hepatic and metabolic disorders.

**Table 2:** Influence of flavonoids and gut microbiota on hepatic energy regulation.

**Study type**	**Biological model**	**Compounds**	**Method/dose**	**Effects**	**References**
Animal model	Male Sprague-Dawley rats	Myricetin	HFD + myricetin supplementation (100 mg/kg body weight daily) for 12 weeks	↓ Hepatic lipid synthesis; ↓ inflammation; modulated gut microbiota composition; ↑ fecal butyric acid levels; improved gut barrier function; activated the AMPK signaling pathway.	(79)
Animal model	Germ-free mice	Quercetin	Gut microbiota transplantation from donors with varying responses to HFD and quercetin; quercetin supplementation at 50 mg/kg body weight daily	Modulated gut microbiota by increasing *Akkermansia spp*. and SCFA levels; ↓ inflammation by downregulating TNF-α, IL-6, and TLR-4; counteracted endotoxaemia; ↓ hepatic triglyceride accumulation; protected against NAFLD progression	(80)
Animal model	Male C57BL/6J mice	Quercetin	0.05% quercetin supplementation in HFD for 6 weeks	↓ Liver weight; ↑ relative abundance of *Akkermansia* in gut microbiota; ↓*Firmicutes*/*Bacteroidetes* ratio; ↓ hepatic expression of SREBF1, PPAR-α, CYP51, and SCD1.	(81)
Animal model	Male C57BL/6J mice	Galangin	Chronic alcohol consumption plus oral administration of galangin at 50 mg/kg body weight daily for 8 weeks	↓ Oxidative stress and NLRP3-mediated inflammation; modulated gut microbiota composition by increasing *Akkermansia* abundance; improved intestinal barrier function; ↑ levels of SCFAs; alleviated liver pathological damage.	(82)
Animal model	Male Wistar rats	Silymarin	1% silymarin in HFD for 12 weeks	Modulated gut microbiota composition; ↑ microbial production of vitamin B12; ↓ hepatic lipogenesis; ↑ fatty acid oxidation.	(84)
*In vitro*	HepG2 cells	Apigenin	Co-treatment with 250 μM palmitic acid and apigenin (10, 20, or 40 μM) for 24 h	↓ Total cholesterol and triglyceride levels; reduced intracellular lipid accumulation; ↑ phosphorylated-AMPK levels; ↓ expression of SREBP-1, SREBP-2, FAS, and HMGCR in a concentration-dependent manner.	(85)
Animal model	Mice infected with *S. japonicum*	Total flavonoids of litchi seed (TFL)	TFL administered orally at doses of 50 and 100 mg/kg daily for 8 weeks	Improved liver pathology by reducing granulomatous lesions; ↓ expression of α-SMA, Collagen I, and Collagen III in liver tissues; modulated gut microbiota composition altered by *S. japonicum* infection.	(87)

↑, increase; ↓, decrease; AMPK, AMP-activated protein kinase; CYP51, cytochrome P450 family 51 subfamily A member 1; FAS, fatty acid synthase; HFD, high-fat diet; HMGCR, 3-hydroxy-3-methylglutaryl-CoA reductase; IL-6, interleukin 6; NAFLD, non-alcoholic fatty liver disease; NLRP3, NOD-like receptor pyrin domain-containing 3; PPAR-α, peroxisome proliferator-activated receptor α; SCD1, stearoyl-CoA desaturase 1; SCFAs, short-chain fatty acids; α-SMA, α-smooth muscle actin; SREBP, sterol regulatory element-binding protein; SREBF1, sterol regulatory element-binding transcription factor 1; TLR, toll-like receptor; TNF-α, tumor necrosis factor α.

Non-alcoholic fatty liver disease (NAFLD) is the most common form of liver disease and is closely associated with the increasing prevalence of obesity (77). Intestinal dysbiosis plays a significant role in this process, contributing to the development of diet-induced obesity. The portal vein serves as the primary communication route between the intestine and the liver, facilitating the transfer of metabolites and microbial compounds. When the intestinal barrier is compromised, pathogens, bacterial factors, and their metabolites can translocate to the liver via the hepatic-intestinal circulation. This process triggers inflammatory and immune responses, leading to liver damage (78).

Myricetin supplementation has demonstrated a significant protective effect against NAFLD induced by a HFD in rats, markedly delaying disease progression. This effect was associated with the modulation of intestinal microbiota composition, particularly an increase in the abundance of butyric acid-producing bacteria, such as *Bacteroides* sp. (79). Furthermore, the results indicated greater activation of the AMPK pathway, as evidenced by increased AMPK phosphorylation and a reduction in the levels of acetyl-CoA carboxylase (ACC) and 3-hydroxy-3-methylglutaryl-CoA reductase (HMGCR), both essential enzymes in hepatic lipid synthesis (79). Surprisingly, the rats exposed to HFD for 12 weeks did not develop overweight and showed changes in food consumption. Although several metabolic parameters altered by obesity were present, the animals failed to show signs of overweight and obesity. Quercetin modulated gut microbiota and the progression of NAFLD in germ-free mice that received fecal microbiota transplantation from donors fed a HFD (80). Additionally, quercetin supplementation promoted the growth of beneficial bacteria, such as *Akkermansia spp*., and increased the levels of SCFAs, including acetate and butyrate, which are recognized for their beneficial effects on gut and liver health (80). Furthermore, a significant reduction in the hepatic expression of pro-inflammatory genes, such as *Tnf-*α and *Il-6*, was observed, helping to partially counteract HFD-induced endotoxemia (80). Quercetin also markedly reduced the activation of TLR-4, a key mediator of liver inflammation. Finally, quercetin led to a partial reduction in hepatic triglyceride accumulation, while also exerting a protective effect against the development of histological changes characteristic of NAFLD (80). In a study conducted by Tan et al. (81), the administration of quercetin to mice fed a HFD resulted in a reduction in total hepatic lipids. Furthermore, an increase in the relative abundance of the bacterial genera *Akkermansia, Bacteroides, Marvinbryantia*, and *Romboutsia* was observed, while the abundance of *Blautia, Clostridium sensu stricto 1, Erysipelatoclostridium, Lactobacillus*, and *Turicibacter* decreased (81). Additionally, the mRNA expression of hepatic genes involved in cholesterol and bile acid synthesis, including SREBF (sterol regulatory element binding transcription factor), PPAR-α, CYP51 (cytochrome P450, family 51, encoding lanosterol 14α-demethylase), and SCD1 (stearoyl-coenzyme A desaturase 1), was downregulated (81). These findings indicate that quercetin protects against HFD-induced hepatic alterations by simultaneously modulating lipid metabolism and gut microbiota composition (81).

Galangin, a flavonol widely used in the treatment of conditions such as the common cold, has demonstrated significant hepatoprotective properties (82). In a study involving mice with alcohol-induced liver injury, the administration of galangin resulted in a marked attenuation of liver damage. The hepatoprotective effects of galangin were evidenced by the restoration of hepatic redox balance, as reflected in the reduction of antioxidant enzyme levels, including superoxide dismutase (SOD), glutathione peroxidase (GSH-Px), and total antioxidant capacity (T-AOC) (82). Additionally, galangin exhibited anti-inflammatory properties by reducing the activation of the NLRP3 inflammasome, a key mediator of alcohol-induced inflammation. Analysis of the gut microbiota revealed that galangin administration positively influenced bacterial composition (82). There was an increase in the abundance of *Bacteroidetes* and a reduction in the *Firmicutes/Bacteroidetes* ratio. Furthermore, galangin significantly enhanced the levels of *Akkermansia*, a beneficial bacterium known for its role in maintaining intestinal barrier integrity and reducing liver inflammation. This increase was associated with a reduction of *Bilophila*, a bacterial genus linked to inflammation and gut dysbiosis. Another noteworthy finding was the increase in the levels of SCFAs, including acetic acid, propionic acid, and butyrate (82).

Silymarin, a mixture of flavonolignan compounds, is widely recognized for its hepatoprotective properties. However, its limited bioavailability presents a challenge to its therapeutic efficacy (83). A study conducted by Sun et al. (84) investigated the effects of silymarin supplementation in an experimental model of HFD-induced hepatic steatosis in rats. Silymarin improved lipid metabolism and liver function, as evidenced by the reduction in plasma levels of alanine transaminase (ALT) and aspartate transaminase (AST), as well as of inflammatory markers IL-6 and TNF-α in the liver. These effects were associated with beneficial changes in IM along with an increase in bacterial production of vitamin B12, a factor that has been associated with improved lipid metabolism (84).

In HepG2 cells, exposure to palmitic acid induced excessive lipid accumulation, while subsequent treatment with different concentrations of apigenin led to a significant reduction in triglyceride, total cholesterol, and intracellular lipid levels (85). Furthermore, apigenin increased AMPK phosphorylation, while concentration-dependently reducing the expression of HMGCR, SREBP-1, fatty acid synthase (FAS), and SREBP-2. These findings suggest that apigenin may modulate hepatic lipid metabolism by promoting AMPK activation and reducing lipid synthesis (85). However, this hypothesis must be tested *in vivo* studies.

Hepatic fibrosis is characterized by the excessive accumulation of extracellular matrix proteins, leading to the formation of scar tissue, which can result in permanent liver damage and chronic inflammation (86). Schistosomiasis, caused by *Schistosoma japonicum* (*S. japonicum)* infection, is one of the key factors contributing to the development of this condition. Specific pathogen-free mice were infected with *S. japonicum* and subsequently treated with total lychee seed flavonoids (TFL). A formulation containing bioactive compounds such as quercetin, catechin, and methyl 5-O-p-coumaroylquinic acid (87). The treatment significantly improved pathological changes in the liver by reducing the expression of α-SMA (alpha-smooth muscle actin), collagen I, and collagen III, which are key markers of hepatic fibrosis (87). Interestingly, TFL contributed to the partial restoration of intestinal microbiota balance by decreasing the abundance of pathogenic bacteria. TFL also promoted the growth of beneficial bacteria, such as *Lactobacillus* and *Bifidobacterium*, which play a crucial role in regulating inflammation and maintaining hepatic homeostasis (87).

Given the liver's central role in the body's metabolic flexibility, proper functioning of the gut-liver axis is essential for metabolic health. The data presented in this section are more homogeneous than in other tissues, which may be due to the close collaboration between the gut and liver in metabolic control. Flavonoids exert their effects through mechanisms like those in other tissues. These mechanisms promote lipid metabolism, anti-inflammatory events, regulation of oxidative stress, production of SCFAs, and modulation of the gut microbiota. The data presented demonstrate the interdependence between the two organs, positioning IM as an important mediator of flavonoids' effects on energy metabolism.

## 7 Impact of the interaction between flavonoids and intestinal microbiota on adipose tissue metabolism

The gut-adipose tissue axis is a key component in the regulation of EB in the body (Table 3), as the IM produces metabolites that are transported through circulation to several systems and organs (88). These metabolites include tryptophan-derived molecules, bile acids, components of bacteria (e.g., ligands that bind to toll-like receptors), pattern recognition receptors containing the nucleotide-binding oligomerization domain (NOD), and SCFAs (88).

**Table 3:** Adipose tissue responses to flavonoid-mediated microbiota modulation.

**Study type**	**Biological model**	**Compounds**	**Method/dose**	**Effects**	**References**
Animal model	Male Wistar rats	Anthocyanins	Diet 10/100 g blueberry for 8 weeks	At IM level: *↑ Gammaproteobacteria* *↑ Proteobacteria* *↑ Fusobacteria* *↓ Firmicutes* At WAT level: ↓ TNFα, ↓ IL-1β, ↓ CD11, ↑ PGC-1α, ↑ PPAR-δ At BAT level: –	(91)
Animal model	Male C57BL/6J mice	Gallic acid catechin epicatechin	Diet Grape seed proanthocyanidin 100 or 200 mg/kg for 8 weeks	At IM level: *↓ Firmicutes/Bacteroidetes* *↓ Weissella* *↑ Faecalibaculum* *↑ Bacteroides* *↑ Akkermansia* *↑ Ruminococcus* At WAT level: ↑ PGC-1α, ↑ UCP1, ↑ PRDM16 At BAT level: ↑ PGC-1α, ↑ UCP1	(94)
Animal model	Male C57BL/6 mice	Tangeretin	Diet with tangeretin 100 mg/kg	At IM level: *↓ Firmicutes/Bacteroidetes* *↑ Bacteroides* *↑ Lactobacillus* At WAT level: – At BAT level: ↓ IL-1β, ↓ TNF-α, ↓ FAS, ↓ ACC-1, ↑ PGC-1α, ↑ PPAR-γ, ↑ UCP1, ↑ UCP2, ↑ TMEM26, ↑ CD137, ↑ CIDEA	(95)
Animal model	Male C57BL/6J mice	Isoliquiritigenin	Isoliquiritigenin diet for up to 12 weeks	At IM level: *↑ Parabacteroides goldsteinii* *↑ Akkemansia muciniphila* At WAT level: ↓ TNFα, ↓ MCP-1, ↑ Adiponectin, ↑ F4/80, ↑ CD11c, ↑ CD206 At BAT level: –	(96)
Animal model	Male and female C57Bl6/J mice	7,8-dihydroxyflavone	Diet 1 mg/mL of 7,8-dihydroxyflavone for 12-weeks	At IM level: – At WAT level: – At BAT level: ↑ TrkB, ↑ UCP1, ↑ AMPK, ↑ SIRT1, ↑ PGC1 Only females	(97)
Animal model	Male C57BL/6J mice	Gamma-amino butyric acid (GABA), chlorogenic acid, gallic acid, rutin	Diet 20% mulberry leaves for 13 weeks	At IM level: *↑ Bacteroidetes/Firmicutes* *↑ Akkermansia* *↓ Proteobacteria* At WAT level: – At BAT level: ↑UCP1, ↑ PGC-1α, ↑ PGC-1β, ↑ PPAR-γ, ↑ CPT1α, ↑ C/EBPα, ↑ C/EBPβ	(99)
Animal model	Female C57BL/6J mice	Genistein	Genistein 30 mg/kg/day by gavage for 10 weeks	At IM level: *↑ Ruminiclostridium* *↑ Rikenella* *↑ Clostridium* At WAT level: – At BAT level: ↑ UCP1, ↑ PGC-1α	(100)
Animal model	Male C57BL/6J mice	Naringenin	Naringenin 100 mg/kg by gavage for 12 weeks	At IM level: *↑ Akkermansiaceae* *↑ Akkermansia* *↓ Lachnospiraceae* *↑ Verrucomicrobiales* At WAT level: ↑ PGC-1α, ↑ CPT1α, ↑ ATGL, ↑ HSL, ↑ MCAD*, ↑* TFAM, ↑ NRF1, ↑ NRF2, ↑ CD137, ↑ HOXC8, ↑ TBX1, ↑ UCP1 At BAT level: –	(101)
Animal model	Male C57BL/6 mice	3′-Hydroxydaidzein (OHD) Daidzein (DAI)	Diet with 0.1% DAI (0.5 g DAI in 500 g HFD diet) Or 0.05% 3′-hydroxydaidzein (LOHD, 0.25g 3′-hydroxydaidzein in 500 g HFD diet) HDF supplemented with 0.1% 3′-hydroxydaidzein (HOHD, 0.5 g 3′- hydroxydaidzein in 500 g HFD diet) for 13 weeks	At IM level: *↓ Firmicutes/Bacteroidetes* *↑ Lachnospira* *↑ Verrucomicrobia* *↑ Lactobacillus* *↑ Blautia* *↑ Lachnoclostridium* At WAT level: ↓ Adipocyte size ↑ PRDM16, ↑ UCP1, ↑ PGC1, ↑ p-HSL, TMEM26, ↑ SIRT1, ↑ P-P38, ↑ C/EBP β At BAT level: –	(102)

↑, increase; ↓, decrease; –, not reported; ACC-1, acetyl-CoA carboxylase 1; AMPKα, AMP-activated protein kinase; ATGL, adipose triglyceride lipase; BAT, brown adipose tissue; CD11, cluster of differentiation 11; CD206, cluster of differentiation 206; CD137, cluster of differentiation 137; CD40, cluster of differentiation 40; C/EBPα, CCAAT/enhancer-binding protein α; C/EBP β, CCAAT/enhancer-binding protein β; CIDEA, cell death inducing DFFA like effector A; CITED1, Cbp/P300 interacting transactivator with glu/Asp rich carboxy-terminal domain 1; COX2, cyclooxygenase 2; CPT1α, carnitine palmitoyltransferase 1α; FAS, fatty acid synthase; FGF21, fibroblast growth factor 21; HMGCR, 3-hydroxy-3-methylglutaryl-CoA reductase; HOXC8, homeobox C8; HSL, hormone-sensitive lipase; IL-1β, interleukin 1β; IM, intestinal microbiota; MCAD, medium-chain acyl-CoA dehydrogenase; MCP-1, monocyte chemoattractant protein-1; NRF1, nuclear respiratory factor; NRF2, nuclear respiratory factor 2; p-AMPKα, phosphorylated AMP-activated protein kinase α; PGC-1α, peroxisome proliferator-activated receptor gamma coactivator 1α; P-P38, protein p 38; PPAR-δ, peroxisome proliferator-activated receptor δ; PPAR-γ, peroxisome proliferator-activated receptor γ; PRDM16, PR domain containing 16; SIRT1, sirtuin 1; SREBP1c, sterol regulatory element-binding transcription factor 1c; TBX1, T-box transcription factor 1; TFAM, mitochondrial transcription factor A; TMEM26, transmembrane protein 26; TNF-α, tumor necrosis factor α; TrkB, tropomyosin receptor kinase; UCP1, uncoupling protein 1; UCP2, uncoupling protein 2; WAT, white adipose tissue.

Various SCFAs produced by bacteria, such as *Akkermansia muciniphila* (*A. muciniphila*), are important regulators of intestinal activity as well as the main signaling molecules in this axis (88, 89). In the context of obesity, gut microbiota dysbiosis caused by HFD consumption is ameliorated by supplementation with flavonoids found in functional foods, as shown by several studies (90). Blueberry powder and grape seed extract, rich in proanthocyanins, increased serum and colonic SCFA concentration, particularly acetate, propionate, and butyrate (91, 92). Blueberry supplementation increased the expression of the SCFA receptor FFAR2 (91). This study used rats exposed to a diet with 40% calories from fat. The diet did not induce significant differences in *Bacteroidetes* and *Firmucutes*, as observed in models with a higher calorie content from fat (60%). However, blueberry treatment modified the gut microbiota, increasing the presence of bacteria from the *Lactobacillales* order and *Proteobacteria* phyla (91). The latter, particularly *Gammaproteobacteria*, has been associated with metabolic improvements in rats undergoing Roux-en-Y gastric bypass (93). Consistent with this, treatment of HFD-exposed mice with 200 mg/kg of a grape seed proanthocyanidin extract for 8 weeks normalized the colonic *Firmicutes*/*Bacteroidetes* ratio. The treated animals exhibited reduced BW gain and improved HFD-induced insulin resistance (94). The SCFA measurement showed a rise in acetate, propionate, and butyrate because of treatment (94). Similar results were observed in mice exposed to HFD treated with the flavonoid tangeretin. HFD supplemented with 100 mg/kg of tangeretin significantly attenuated the obese phenotype, reducing the *Firmicutes*/*Bacteroidetes* ratio and increasing the abundance of *Lactobacillus*. The authors suggest that these effects are due to a reprogramming of the gut microbiota by the flavonoid, as this compound exhibits poor absorption (95). This reprogramming may have led to an increase in the abundance of SCFA-producing bacteria, which, as has been mentioned, can promote metabolic benefits. However, the study did not report SCFA levels. Alternatively, the effects may have been caused by bacteria metabolizing tangeretin, whose metabolites may be responsible for these effects.

Despite a consensus that SCFAs are involved in flavonoids' metabolic benefits, studies have shown that these compounds can still have metabolic benefits without influencing SCFA levels. In a study conducted in mice exposed to HFD (60% calories from fat), treatment with the flavonoid Isoliquiritigenin for 12 weeks significantly reduced BW gain and epididymal WAT (96). Other outcomes included reduced levels of proinflammatory markers as well as normalized levels of *Firmicutes*/*Bacteroidetes* ratio. The abundance of *Parabacteroides goldsteinii* and *A. muciniphila*, both reported to alleviate obesity and diabetes, was augmented. An improved glucose and insulin response was also observed. All effects were independent of SCFAs, as isoliquiritigenin treatment failed to alter acetate, propionate, and butyrate levels (96). The metabolic benefits depend on changes in IM induced by isoliquiritigenin, as these were replicated in mice exposed to HFD and that received microbiota donated by HFD mice treated with the flavonoid (96).

Different mechanisms, targeting both brown adipose tissue (BAT) and white adipose tissue (WAT), mediate the metabolic effects of the flavonoid-IM interaction. The most mechanisms involve an increase in adaptive thermogenesis, adipose tissue darkening process, adipose tissue mitochondrial biogenesis, as well as a decrease in mitophagy, inflammation, and oxidative stress (12, 90, 95, 97). The proanthocyanidins, found in grape seeds and Chinese laurel, have been shown to induce thermogenesis, browning, β-oxidation, and lipolysis in adipose tissue (94). These effects are revealed by an increase in UCP1 expression, in BAT and WAT, as well as an increase in PGC-1α in WAT (94). This browning process was also observed in a study carried out in the context of functional foods. Mice were simultaneously subjected to HFD and treatment for 10 weeks with Ougan juice, with or without fermentation using *Lactobacillus casei* Lpc37 (98). Both the unfermented and fermented juice significantly decreased BW gain and glucose intolerance, and normalized insulin sensitivity. In addition to increasing SCFA levels, the treatment increased UCP1 in epididymal WAT. The fermented juice induced the most pronounced effects, which could have been achieved through two pathways, either through the production of narigenin or by increasing the colonic abundance of Lactobacillus (98). In another study, a mulberry leave extract, containing gamma-aminobutyric acid (GABA), gallic acid, cholinergic acid, and rutin, was supplemented in a HFD (99). The treatment increased thermogenesis in BAT and induced browning phenotype in WAT by upregulating genes such as PGC-1α, PGC-1β, PPAR-γ, carnitine palmitoyltransferase 1α (CPT-1α), and UCP1 (99). Genistein, an isoflavonoid found in soy, has also been shown to protect against obesity by increasing the expression of the thermogenic genes UCP1 and PGC-1α in BAT (100). Interestingly, suppression of IM inhibited the effect of genistein, suggesting the critical role of gut microbiota in the anti-obesity effects (100). In the same line, naringenin promoted thermogenic activity in BAT and increased SCFA levels in cecum and blood serum (101). It seems that both the metabolites of flavonoids and the flavonoids themselves have a regulatory effect on obesity. A study evaluated in mice the metabolic effects of 0.1% of daidzein or its metabolite3′-hydroxydaidzein, at different doses (0.05 and 0.1%), offered in a HFD (102). Data showed increased levels of WAT browning markers such as C/EBP β (CCAAT/enhancer-binding protein beta), SIRT1, and P-P38 (P38 protein), but only the group treated with the metabolite showed an increase in PRDM16 (PR domain containing 16), which is a regulator of adipose tissue cell metabolism (102).

HFD-induced obesity is associated with low-grade systemic inflammation, endotoxemia, and an increase in intestinal wall permeability. A study showed that blueberry powder supplementation reduced intestinal wall permeability by increasing the expression of mucin 2 (MUC2) and β-defensin 2 (DEFB2). The treatment also reduced the expression of pro-inflammatory cytokines such as TNF-*a* and IL-1β in visceral fat (91). Tangeritin showed the ability to reduce the expression of pro-inflammatory cytokines such as TNF-*a* and IL-1β in WAT (95). Another study shows the anti-inflammatory effect of isoliquiritigenin by down-regulating inflammatory marker genes and macrophage activity TNF-α, MCP-1 and CD11 in WAT (96). Interestingly, this flavonoid increased the expression of adiponectin, an anti-inflammatory hormone. In addition, isoliquiritigenin promoted the expression of MUC2 in LS174T cells and TJP1 (gene encoding tight junction proteins) in the colon (96).

Adipose tissue is one of the organs most studied due to its direct relationship with obesity. A wide range of studies has sought to understand in detail the effects of flavonoids on adipocyte metabolism, with the aim of developing flavonoid-based therapies for obesity. These compounds offer the advantage of not posing a risk of toxicity. Thus, the impact of flavonoids on WAT and BAT has been extensively studied. The studies presented in this section clearly show the role played by IM as an intermediary in this impact. The evidence clearly shows that flavonoids and the microbiota interact, producing metabolites released by bacteria (e.g., SCFAs) or produced from the microbial metabolism of flavonoids (e.g., 3′-hydroxydaidzein). These metabolites have a significant impact by inducing thermogenesis, browning, and anti-inflammatory processes. The loss of flavonoid effects in animals subjected to microbiota depletion indicates a causal role of IM in the metabolic benefits caused by these phytonutrients. However, further studies are needed to establish the most suitable conditions for their use in combating obesity.

## 8 Impact of the interaction between flavonoids and intestinal microbiota on hypothalamic regulation of metabolism

The gut-brain axis has attracted interest in understanding how it works, as it has been shown to be involved in the development of neurodegenerative and metabolic diseases (103–105). The hypothalamus, particularly the arcuate nucleus (ARC), is a center that regulates EB because it contains specialized neurons detecting the body's energy levels. Such neurons compose the melanocortin, bile acid-signaling, and thyroid systems, playing an important role in the regulation of EB (106–108).

It was only recently that the mechanisms by which IM regulates the ARC activity, or *vice versa*, have begun to be elucidated. The understanding of how hypothalamic neurons detect IM-derived signaling molecules or induce gut microbiota plasticity is still developing. Recent studies have provided insight into these unidentified mechanisms. First, a study conducted in mice recently reported the expression of the NOD2 (nucleotide-binding oligomerization domain 2) receptor (recognizing bacterial wall-derived components) in neurons of the ARC (109). Simulation of NOD2 with a ligand (muramyl dipeptide) in GABAergic neurons, food intake was decreased. Conversely, hyperphagia, BW gain, and dysregulation of thermogenic processes were observed when NOD2 was ablated in hypothalamus (109). It is worth mentioning that the treatment was given orally, indicating that the ligand reached the ARC. An additional experiment suggested that IM was involved in these effects, as the elimination of microbiota by antibiotic treatment inhibited NO2′s control of food intake (109).

SCFAs, bile acids, and cytokines may be involved in IM signaling to the hypothalamus as another mechanism. SCFAs and secondary bile acids can activate FFAR2 and the membranal G protein-coupled Takeda receptor 5 (TGR5), respectively, to induce GLP-1 and peptide YY (PYY) secretion from L cells of the distal gut (103, 110). Once secreted both GLP-1 and PYY can reach hypothalamus and regulate EB (111). Bile acids have recently been shown to play a critical role in the hypothalamic regulation of EB. It has recently been shown that the activation of TGR5 in the ARC by bile acids, or synthetic agonist, promotes energy expenditure and weight loss (106, 112). Thus, TGR5 in the hypothalamus could mediate the effects of bile acids, produced by IM, on EB. Microglia-depending hypothalamic inflammation caused by HFD is a crucial factor in the development of obesity (113). In conditions of obesity, pro-inflammatory microbiota could favor the trafficking of monocytes to the hypothalamus, which has been shown to be mediated by peripheral inflammation (114). According to this hypothesis, the microbiota's diversity affects the maturation and function of microglia, which is regulated through FFAR2 (115).

While evidence of the mechanisms involved in the communication of IM to the ARC is limited, evidence of the mechanisms in the opposite direction is even scarcer. However, a recent study provided concrete evidence of the regulation of IM by proopiomelanocortin (POMC) neurons. Combining genetic, molecular, and behavioral tools, Toledo et al. (116) demonstrated that chemogenetic, or leptin-mediated, activation of POMC neurons in the ARC induces rapid (2 h) modulation of duodenal microbiota composition. These effects were observed in fasted mice treated intracerebroventricularly, indicating that the changes were elicited by the ARC without interference from food intake. The same treatment in mice with leptin resistance due to HFD exposure failed to replicate the effects, indicating the need for intact leptin signaling (116).

The interaction between flavonoids and the IM-hypothalamus axis has not yet been elucidated. The flavonoid quercetin improved the intestinal microbiota by increasing the abundance of *Lactobacillus*, which promoted the synthesis of bile acids, particularly ursodeoxycholic acid (UDCA) and lithocholic acid (LCA), in a mouse model exposed to HFD (44). Acetate is a product of the fermentation of dietary fiber performed by flavonoid-promoted beneficial IM. As previously mentioned, this SCFA is a crucial regulator of the gut-brain axis. It has also been shown to have the ability to modulate hypothalamic regulation of food intake and BW. Mice treated with acetate exhibited reduced BW gain and food intake, due probably to POMC signaling, as enhanced mRNA expression was observed 30 min after administration (117). Consistent with this, reduced BW gain was observed in rats treated with acetate. Interestingly, acetate normalized peripheral and hypothalamic oxidative stress as well as cytokine expression, two conditions associated with obesity (118). Another study conducted in mice suggests that acetate reduces food intake by reducing orexin neuronal activity. Such an effect requires, in part, leptin homeostasis, as *ob*/*ob* mice exhibited lower response to acetate (119). The mice also showed increased expression of POMC, even in the presence of orexin, which is an inhibitor of POMC neurons (119). Acetate plays a significant role in the interaction between IM and hypothalamus induced by flavonoids. However, a positive correlation between acetate levels and obesity has also been found in preclinical (120) and clinical studies (121). Gastrointestinal hormones and peptides are significant in the hypothalamic regulation of EB. Cholecystokinin (CCK) regulates food intake, as ARC neurons are known to express their receptors (122). Catechin and epicatechin increased the release of CCK in the duodenum (123) and the release of this peptide was found to be positively correlated to the composition of the gut microbiota (122).

Based on the study by Toledo et al. (116), which showed changes in IM after POMC neurons modulation, flavonoids might modulate IM by acting in the ARC. A range of studies has reported metabolic effects of flavonoids on hypothalamic EB regulation (124). Kaempferol administered orally increased POMC expression (125) and its intracerebroventricular injection in obese mice reduced BW (126), which is regulated by POMC. Another study investigated the effects on BW of an avocado seed extract, with a high content of epicatechin, quercetin, and kaempferol. Mice with obesity were treated with oral avocado seed extract, observing reduced BW and enhanced expression of deiodinase 2 (Dio2) in the ARC (127).

Although there is clear evidence of an interaction between the hypothalamus and IM in regulating EB, studies are still limited. The role of flavonoids in this interaction is highly speculative, requiring additional research to demonstrate their role. The research on the IM-hypothalamus axis' role in energy metabolism regulation is promising. IM products, including bile salts, SCFAs, and bacterial components, might be induced by flavonoid treatment. Given that the hypothalamus plays a central role in the regulation of energy homeostasis and BW, studying the mechanisms of crosstalk with IM is of great importance in the fight against metabolic diseases such as obesity and diabetes.

## 9 Conclusion and perspectives

Modern eating habits are characterized by the consumption of high-calorie diets, which lead to dysbiosis, inflammation, and energy balance dysregulation, contributing to the development of obesity and obesity-related diseases. The lack of effective long-term pharmacological therapies has led to the search for dietary solutions. Accumulating evidence places flavonoids as leading candidates to mitigate the devastating effects of obesity. Despite a consensus in the scientific community regarding the plausible use of these phytonutrients to treat metabolic disorders, this has not been fully implemented in practice. One reason for this lack of success is the complexity of the mechanisms involved in their bioactivity, which vary depending on the specific flavonoid. A complete understanding of these mechanisms is essential so that their therapeutic potential can be clinically employed. This work reviewed preclinical research existing in the literature investigating the mechanisms involved in the effects of flavonoids on the gut microbiota and their metabolic consequences in metabolically important tissues. The gut microbiota is highly relevant, as it is a determining factor in the protection against metabolic diseases such as obesity and diabetes, as well as digestive diseases such as colitis (128). The reviewed literature clearly shows that flavonoids exert metabolic benefits through modulation of IM, promoting the abundance of beneficial bacteria and reducing the presence of pathogenic bacteria. Compounds such as SCFAs, bile acids, and bacterial components produced by a healthy gut microbiota modulate energy homeostasis in metabolically important tissues such as skeletal muscle, liver, adipose tissue, and the hypothalamus. Bioactive metabolites produced by the gut microbiota from the degradation of flavonoids represent another mechanism by which these phytonutrients regulate energy homeostasis. Despite the consistency of the results, they were obtained in preclinical studies of rodents under controlled conditions. This makes it difficult to extrapolate the results to humans, who exhibit enormous variability in genetic background, dietary habits (then differences in IM composition), pathophysiology of metabolic disorders, sleep patterns, and other factors. Extrapolating the results to humans is challenging due to the significant variability in genetic background, dietary habits, pathophysiology of metabolic disorders, sleep patterns, and other factors. Several flavonoids have been co-administered with anti-obesity drugs in humans, showing interesting benefits (129). However, the results have been variable due to the different mechanisms of action elicited by flavonoids and the variability of the studied population. The presence of discordant or poorly reproducible results highlights the need for further studies conducted under controlled experimental conditions to help establish the appropriate procedure for the use of flavonoids.
